# Evidence that RASSF1C Stimulation of Lung Cancer Cell Proliferation Depends on IGFBP-5 and PIWIL1 Expression Levels

**DOI:** 10.1371/journal.pone.0101679

**Published:** 2014-07-09

**Authors:** Mark E. Reeves, Matthew Firek, Shin-Tai Chen, Yousef G. Amaar

**Affiliations:** 1 Surgical Oncology Laboratory, Loma Linda VA Medical Center, Loma Linda, California, United States of America; 2 Musculoskeletal Disease Center, Loma Linda VA Medical Center, Loma Linda, California, United States of America; 3 Department of Surgery, Loma Linda University School of Medicine, Loma Linda, California, United States of America; Institute of Experimental Endocrinology and Oncology 'G. Salvatore' (IEOS), Italy

## Abstract

RASSF1C is a major isoform of the RASSF1 gene, and is emerging as an oncogene. This is in contradistinction to the RASSF1A isoform, which is an established tumor suppressor. We have previously shown that RASSF1C promotes lung cancer cell proliferation and have identified RASSF1C target genes with growth promoting functions. Here, we further report that RASSF1C promotes lung cancer cell migration and enhances lung cancer cell tumor sphere formation. We also show that RASSF1C over-expression reduces the inhibitory effects of the anti-cancer agent, betulinic acid (BA), on lung cancer cell proliferation. In previous work, we demonstrated that RASSF1C up-regulates *piwil1* gene expression, which is a stem cell self-renewal gene that is over-expressed in several human cancers, including lung cancer. Here, we report on the effects of BA on *piwil1* gene expression. Cells treated with BA show decreased *piwil1* expression. Also, interaction of IGFBP-5 with RASSF1C appears to prevent RASSF1C from up-regulating PIWIL1 protein levels. These findings suggest that IGFBP-5 may be a negative modulator of RASSF1C/ PIWIL1 growth-promoting activities. In addition, we found that inhibition of the ATM-AMPK pathway up-regulates RASSF1C gene expression.

## Introduction

The RASSF1 gene plays an important role in human cancer cell growth and progression. It encodes multiple isoforms, the major ones of which are RASSF1A and RASSF1C. RASSF1A is the most frequently inactivated tumor suppressor in human cancers mainly by means of specific promoter methylation. It inhibits cell growth and migration, and promotes apoptosis. On the other hand, the RASSF1C isoform is well expressed in the majority of human cancers, and appears to function as an oncogene. In contrast to RASSF1A, it promotes cancer cell proliferation and migration, and attenuates apoptosis [1]–[13]. Thus, the RASSF1 gene appears to play an important dual role in cancer, functioning alternatively as a tumor suppressor and as an oncogene [1]–[15]. Consistent with this concept, recent studies show that the expression of RASSF1C is up-regulated in human lung carcinoma tissue compared to matched normal tissues, and is associated with cancer progression and poor prognosis [13]. In addition, RASSF1C over-expression (but not RASSF1A over-expression) in human cancer cells enhances accumulation of the β-catenin oncogene, a key player in the Wnt signaling pathway, leading to increased transcriptional activation and cell proliferation [16].

We have previously shown that over-expression of RASSF1C up-regulates (and silencing of RASSF1C down-regulates) the expression of PIWIL1, a stem cell self-renewal gene [12]. The Piwil gene family is a subfamily of the argonaute proteins that plays a central role in stem cell self-renewal, gametogenesis, and transcriptional gene silencing in a wide variety of species. The argonaute proteins bind small RNAs and they are characterized by amino terminal (N), PAZ (Piwil-Argonaute-Zwille), MID (middle), and PIWI domains [17]. In humans, three Piwil (Piwil 1 (also called Hiwi), Piwil2, and Piwil3) genes have been identified. Piwi protein expression profiles have recently received much attention for their potential functional involvement in oncogenesis in a variety of human cancers and Piwil1 and Piwil 2 have been shown to be independent prognostic factors in gastric cancer [17]–[19]. The PIWIL proteins and their interacting small RNAs (piRNAs) may play a role in tumorigenesis through increasing gene methylation and silencing of cyclin dependent kinase inhibitors and tumor suppressors. The PIWIL proteins and their interacting small RNAs (piRNAs) may play a role in tumorigenesis through increasing gene methylation and silencing of cyclin dependent kinase inhibitors and tumor suppressors [17]–[19]. Recent studies show that over-expression of PIWIL1 promotes sarcomagenesis and down-regulates a number of tumor suppressors, including insulin-like growth factor binding protein 5 (IGFBP-5) [20]. IGFBP-5 is a member of the IGF binding protein family involved in the regulation of the mitogens IGF I and II. IGFBP-5 is critically important in human cancer progression [21]; and we have previously shown that RASSF1C is a binding partner of IGFBP-5 [20].

Thus, we wanted to determine if RASSF1C mediates its effects on cancer cells through interactions with IGFBP-5 and PIWIL1. In order to do this, we designed experiments to determine the effects of RASSF1C on lung cancer cell proliferation, migration and tumor sphere formation. Because the anti-cancer agent, betulinic acid (BA), has been shown to down-regulate PIWIL1 gene expression [22], we studied the effects of BA and RASSF1C/IGFBP-5 interaction on PIWIL1 gene expression and β-catenin protein levels. We found that RASSF1C promotes cancer cell migration and tumor sphere formation, and reduces the inhibition of proliferation by BA. In addition, interaction of IGFBP-5 with RASSF1C prevented RASSF1C-mediated up-regulation of PIWIL1. Lastly, silencing of PIWIL1 gene expression decreased β-catenin protein levels, indicating that PIWIL1 may contribute to Wnt signaling. Thus, RASSF1C, IGFBP-5, PIWIL1, and the Wnt pathway could function together as a new axis that impacts lung cancer cell growth and progression.

## Materials and Methods

### Cell culture

The human lung cancer cell lines NCI-H1299 and A549 were obtained from American Type Culture Collection (Manassas, VA). A549 is RASSF1A negative, p16 negative, and p53 positive while NCI-H1299 is RASSF1A negative, p16 negative, and p53 negative. Cell culture was carried out as recommended by ATCC.

### Gene expression vectors

RASSF1C, IGFBP-5, and RASSF1A were over-expressed in human lung cancer cells using inducible Murine Leukemia Virus (MLV)-based retroviral vectors as previously described [12].

### Cell number count

NCI-H1299 cells stably transduced with MLV-backbone (BB) and MLV-HA-RASSF1C (1C) were treated with 1 µg/ml doxycycline for 72 h, and then cells were counted using a hemocytometer. The experiments were repeated at least 3 times.

### Alamar Blue assay

Cell proliferation/viability was measured by the Alamar Blue assay as previously described [11], [12]. The experiments were repeated at least 3 times and data was analyzed using t-test.

### *In vitro* cell invasion assay

The BD BioCoat Matrigel Invasion Chamber (BD Biosciences, Bedford, MA) was used to carry out lung cancer cell invasion assays. NCI-H1299-BB (control) or NCI-H1299-1C (over-expressing RASSF1C) cells were seeded at a density of 25,000 cells in the Matrigel chambers and grown in serum-free RPMI-1640 for 24 h. The chambers containing the NCI-H1299-BB and NCI-H1299-1C cells were then transferred to the well containing medium supplemented with 10% calf bovine serum for 24 h. The cells on the lower surface of the membrane were fixed with methanol and stained with 1% toluidine blue. The stained membranes were photographed through the microscope and invading cells were counted. The experiments were repeated at least 3 times.

### Tumor sphere formation

A CD133 MicroBead Kit (Miltenyi Biotec, Auburn, CA) was used to isolate A549 side population (SP, CD133^+^, cancer stem cells) and non-SP (CD133^-^) cells using a using flow cytometry protocol from the supplier. The SP cells were isolated from A549 transduced with either control MLV-backbone (A549-BB) or MLV-HA-RASSF1C (A549-1C). SP and non-SP cells were then incubated in serum free media supplemented with EFG (20 ng/ml) and FGF (10 ng/ml) to promote SP cells to form tumor spheres for three wk. Tumor spheres were photographed, collected, and plated on media supplemented with 10% calf serum, and cells were grown to 70% confluency. Then cells were used for Western blot analysis to check for expression of exogenous HA-RASSF1C.

### RNA isolation and RT-PCR analysis

Total RNA from human lung cancer cell lines was isolated from cultures and reverse transcriptase (RT)-PCR was performed using PIWIL1 gene-specific primers as previously described [12]. PCR was carried out using the HotStart master mix (Qiagen, Valencia, CA), and the PCR reactions were run with the following conditions: 95°C for 15 min, 95°C for 1 min, 60°C for 30 sec, and 72°C for 30 sec for 35 cycles. Amplification of cyclophyllin using gene specific PCR primers was used as a loading control. The RT-PCR reactions were carried out in triplicates and the fold change was calculated using the 2^−ΔΔCT^ method [23]. The RT-PCR runs were repeated at least 3 times.

### Betulinic acid (BA) treatment

BA (Enzo Life Sciences, New York, NY) was dissolved in DMSO at 5 mg/ml. A549 and NCI-H1299 cells were plated at 5000 cell/well in 96-well plates. Next day, the cells were treated with 25 µg/ml BA for 24 h. Cell proliferation was then assayed using the Alamar Blue assay. The experiments were repeated at least 3 times.

### Silencing of Piwil1 expression in lung cancer cells

Lung cancer cells (NCI-H1299) were plated at 5000/well in 96–well plates 24 hr. before infection and cells were infected with Mission Non-Target shRNA Control Transduction Particles or with multiple Mission Lentiviral shRNA Transduction Particles (NMID: NM_004764) for silencing RASSF1C (Sigma, St. Louis, MO) as previously described [14]. Cells were treated with polybrene (Sigma, ST. Louis, MO) for two hours before adding Lentiviral particle at a Multiplicity of Infection (MOI) of 5. Knockdown validation of *piwil1* expression was assessed by Western blot and qRT-PCR using RASSF1C antibody and RASSF1C specific primers, respectively. The experiments were repeated at least 3 times.

### Western blot analysis

Western blot analysis was carried out using the Odyssey Infrared System (LI-COR Biosciences, Lincoln, NE) and anti-β-catenin antibody #9582 (Cell Signaling, Danvers, MA), anti-PIWIL1 antibody ab12337 (Abcam, Cambridge, MA), anti-HA antibody clone 16B12 (Covance, Berkeley, CA), monoclonal beta actin antibody AC-74 (Sigma, St. Louis, MO), and fluorescently-labeled secondary antibodies IRDye 680RD Infrared Dye (LI-COR Biosciences, Lincoln, NE). The experiments were repeated at least 3 times. Protein levels were normalized to actin levels (the loading control) with standard deviations.

### Transcriptome PCR Array screen

SureFind Transcriptome PCR Array was obtained from Qiagen (Catalog No. 336611. It consisted of 90 cDNA samples derived from the breast cancer cell line MCF7 treated with 90 different chemical inhibitors that regulate various signaling pathways. The PCR array was screened with RASSF1C gene specific primers. Data analysis was performed by importing the Ct values obtained into the SureFind Transcriptome PCR Array Data Analysis Software at http://www.sabiosciences.com/tpadataanalysis.php (Qiagen). The chemicals identified as RASSF1C gene expression regulators were used to treat lung cancer cells to confirm their effects on RASSF1C gene expression using RT-PCR.

### Dorsomorphin and Trichostatin A treatment

H1299 lung cancer cells were treated with Dorsomorphin, also known as compound C, (10 µM) or Trichostatin A (10 µM) in serum free media for 24 h before cells were collected for RNA isolation. Control cells were treated with Dimethyl sulfoxide (DMSO). RT-PCR analysis using RASSF1C, PIWIL1, and cyclophyllin gene specific primers was carried out as mentioned above. Dorsomorphin was obtained from Phoenix Pharmaceutical, Inc (Burlingame, CA) and Trichostatin A was obtained from Reagents Direct (Encinitas, CA). The experiments were repeated at least 3 times.

### Statistical Analysis

The t-test was used to calculate the significance of the data.

## Results

### RASSF1C over-expression enhances lung cancer cell migration

**Figure 1:**
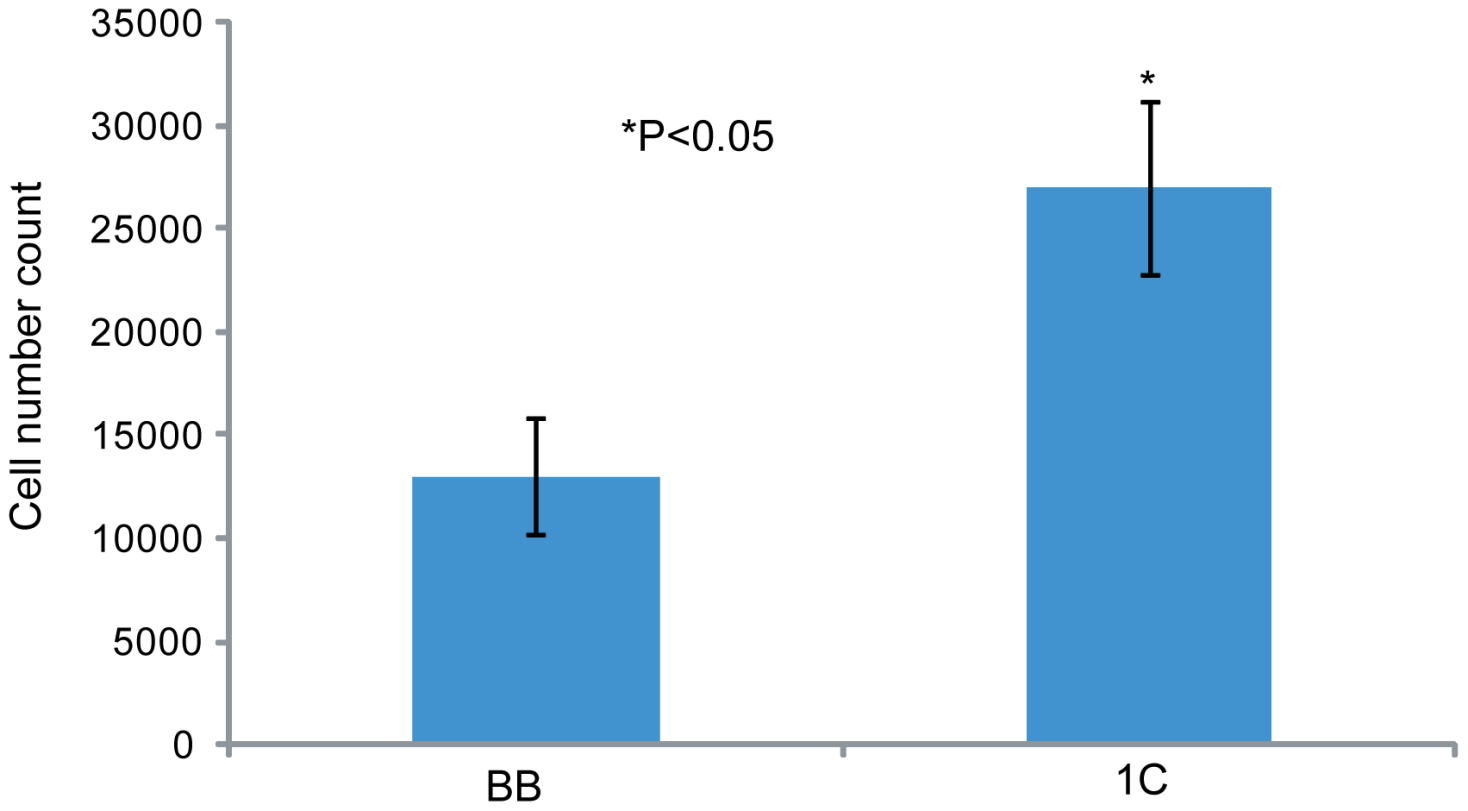
RASSF1C stimulates lung cancer cell proliferation. NCI-H1299 cells stably transduced with MLV-backbone (BB) or MLV-HA-RASSF1C (1C) were treated with 1 µg/ml doxycycline for 72 h, and then cells were counted. RASSF1C over-expression (1C) increased cell proliferation by 2.5 fold compared to control (BB). Data is representative of at least 3 independent experiments, and the values represent the mean±SEM. * P<0.05.

**Figure 2:**
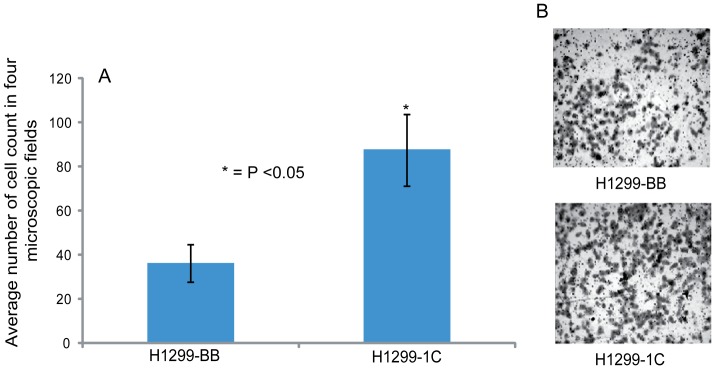
. **RASSF1C promotes lung cancer cell migration.** (A) The BD BioCoat Matrigel Invasion Chamber was used to assess cell invasion /migration of NCI-H1299 cells stably transduced with empty MLV-backbone (BB) or MLV-HA-RASSF1C (1C). Cells treated with doxycycline at 1ug/ml were co-incubated with serum-containing media. After 24 h, the lower sides of the filters were fixed and stained, and cells in four microscopic fields were counted. The average cell number count was plotted. (B) NCI-H1299 cells over-expressing RASSF1C showed a higher number of cells invading the Matrigel chamber and migrating to the other side of the filter compared to control NCI-H1299-BB cells. Data is representative of at least 3 independent experiments, and the values represent the average cell colony count. * P<0.05 as determined by t-test.

The lung cancer cell line NCI-H1299 was transduced with retroviral vector as previously described [12], [13] to create a stable cell line that over-expresses RASSF1C. We found that stable RASSF1C over-expression not only increased lung cancer cell proliferation (Figure 1), which is consistent with our previous findings using transient over-expression of RASSF1C [11], but also promoted cell migration (Figure 2).

### RASSF1C enhances lung tumor sphere formation

**Figure 3:**
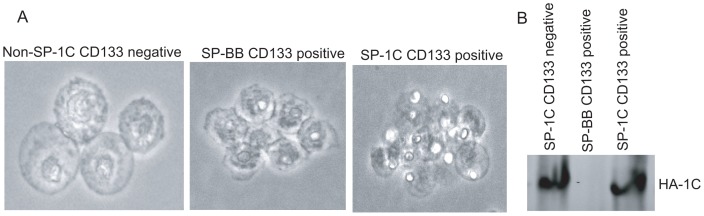
CD133+ cells expressing RASSF1C form tumor spheres more robustly than control cells. (A) Non-SP-1C CD133^-^ , SP-BB CD133^+^ , and SP-1C CD133^+^ cells isolated from the A549 lung cancer cell line were grown in serum free medium supplemented with 10 ng/ml EGF and 20 ng/ml FGF to promote tumor sphere formation for three wk. (B) A549 cells repopulated from tumor spheres were checked for HA-RASSF1C expression using HA-antibody. As expected, SP-1C CD133- and CD133+ expressed HA-RASSF1C, while SP-BB cells did not. Data is representative of at least 3 independent experiments.

It has been demonstrated that CD133-positive cells from the A549 cell line can give rise to tumor spheres and can act as tumor-initiating cells [24], [25]. Thus, we wanted to assess the effect of RASSF1C over-expression on lung cancer tumor sphere formation. We isolated A549 side population (SP, CD133^+^ cells), which display stem cell-like characteristics, and non-SP CD133^-^ cells using CD133 flow cytometry [24], [25]. The SP cells were isolated from A549 transduced with either MLV-backbone (A549-BB) or MLV-HA-RASSF1C (A549-1C). Cells were grown in serum free media supplemented with EFG and FGF to cause SP cells to form tumor spheres (Figure 3A). RASSF1C-SP-CD133^+^ cells formed more and larger tumor spheres compared to control BB-SP-CD133^+^ cells (Figure 3A). In order to show that the tumor sphere progeny still over-expressed RASSF1C, tumor spheres were isolated, grown, and used for Western blot analysis. SP cells did indeed over-express RASSF1C (Figure 3B). All of this suggests that RASSF1C may enhance lung cancer stem cell proliferation, and it is possible that this happens through up-regulation of PIWIL1 gene, a stem cell self-renewal gene [12].

### RASSF1C reduces sensitivity of lung cancer cells to the anti-cancer agent betulinic acid

**Figure 4:**
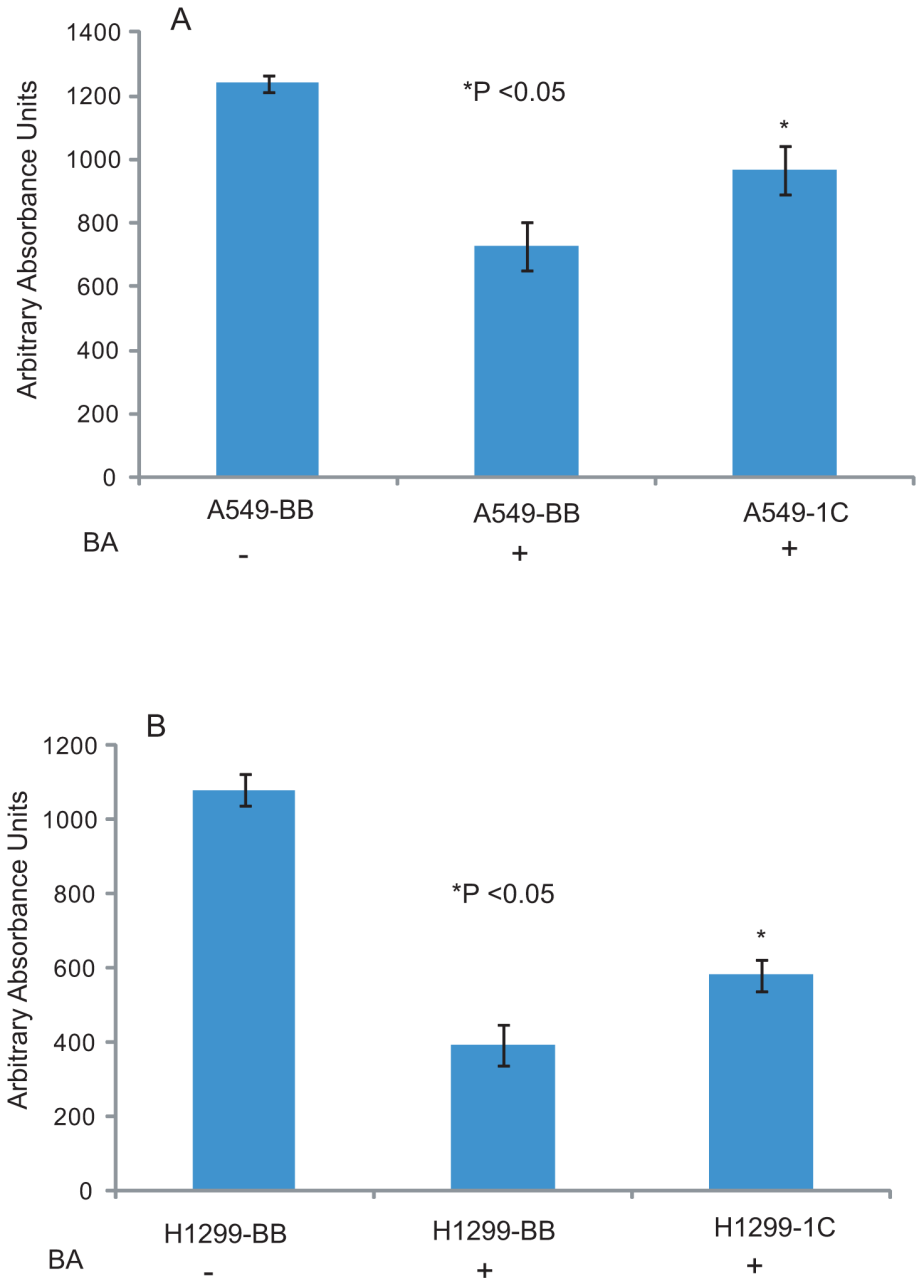
RASSF1C decreases the sensitivity of lung cancer cells to betulinic acid. A549 (A) and NCI-H1299 (B) lung cancer cells over-expressing RASSF1C were treated with betulinic acid (BA) for 24 h. Cells were then assayed for cell viability/proliferation. RASSF1C over-expression significantly reduced the sensitivity of cells to BA. Data is representative of at least 3 independent experiments, and the values represent the mean±SEM. * P<0.05: for 1C compared to BB.

Betulinic acid (BA) is an anti-inflammatory and anti-cancer agent that has been shown to promote apoptosis and inhibit cell proliferation and migration. It does this, at least in part, by down-regulating PIWILI, cyclin B1, cyclin D1, bcl2 and up-regulating bax gene expression in several types of cancer cells, including A549 [22], [26], [27]. We have previously demonstrated that RASSF1C over-expression up-regulates PIWIL1 gene expression and thus we wanted to determine if RASSF1C over-expression would reduce the anti-proliferative effects of BA on lung cancer cells. Our experiments showed that A549 and NCI-H1299 cells over-expressing RASSF1C were less sensitive to BA anti-proliferative effects compared to control cells (Figure 4), further supporting a growth promoting role for RASSF1C in lung cancer cells.

### Effects of BA on PIWIL1 expression in lung cancer cells

**Figure 5:**
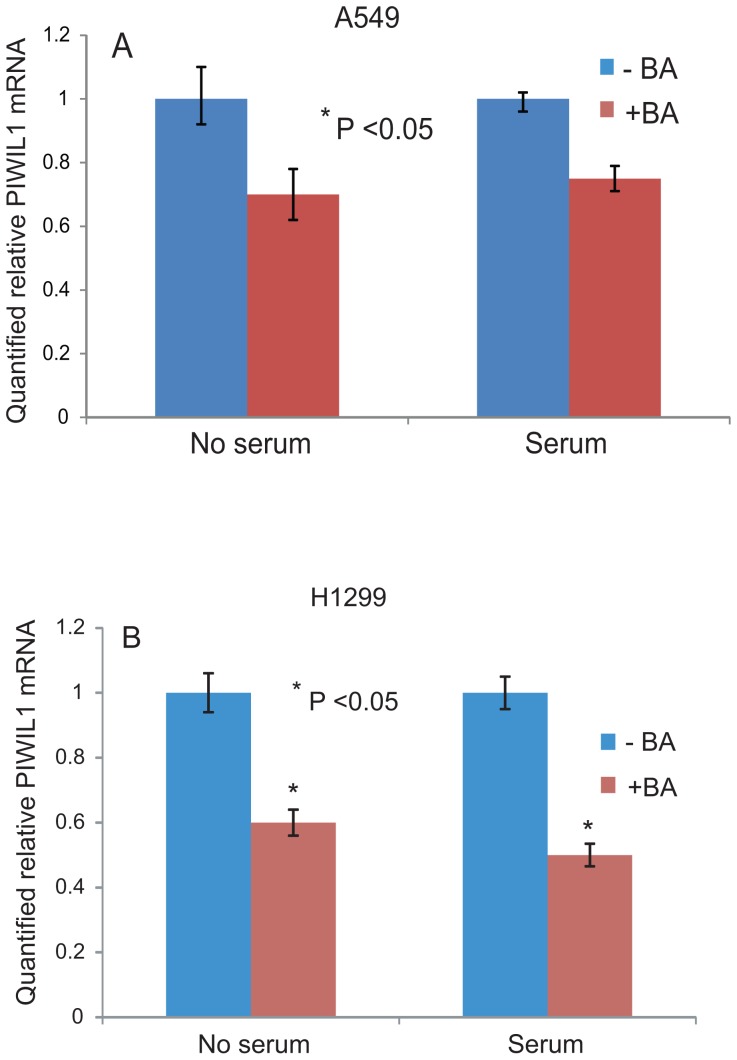
Betulinic acid effects on Piwil1 gene expression. *Piwil1* mRNA expression was assessed in the presence and absence of serum and BA in the lung cancer cell lines A549 (A) and NCI-H1299 (B). RT-PCR data show that *piwil1* expression was down-regulated by BA in the absence and presence of serum. PCR reactions were set in triplicates and Cyclophillin was used as an internal loading control and used to normalize the relative expression levels using the 2^−ΔΔ^ method (26). RT-PCR reaction were run at three independent times.

It has been reported that BA treatment of human gastric adenocarcinoma AGS cells down-regulates PIWIL1 gene expression [26]. Hence, we wanted to assess the effect of BA on PIWIL1 gene expression in lung cancer cells. RT-PCR data (Figure 5) show that *piwil1* mRNA levels were decreased in A549 and H1299 cells upon BA treatment. The down-regulating effect of BA on *piwil1* mRNA levels in lung cancer cells is consistent with that observed in gastric adenocarcinoma AGS cells [26].

### PIWIL1 knock-down leads to reduction of β-catenin expression

**Figure 6:**
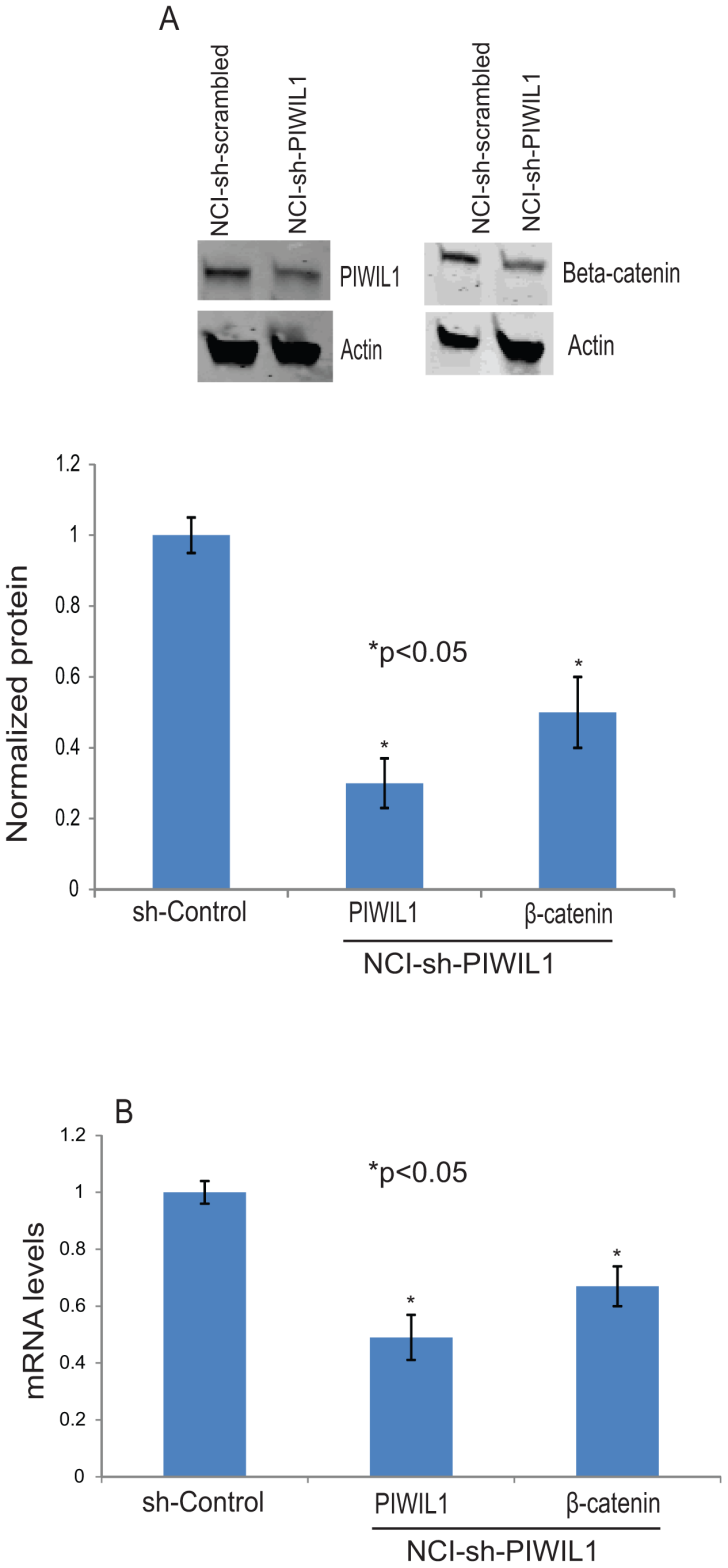
PIWIL1 silencing reduces β-catenin levels in lung cancer cells. Knock-down of *piwil1* expression resulted in a reduction in β-catenin mRNA and protein levels. (A) Western blot analysis of H1299 lung cancer cells infected with Mission Lentiviral-shRNA-control particles (shRNA-cont) and Mission Lentiviral-shRNA-*piwil1* (shRNA-*piwil1*) to silence endogenous *piwil1* expression. Western blot analysis using anti-PIWIL1 antibody shows reduced PIWIL1 protein levels in shRNA-PIWIL1 cells compared to shRNA-cont cells. Silencing of *piwil1* gene expression resulted in a reduction in β-catenin protein levels as determined using β-catenin antibody. Normalized PIWIL1 protein levels to actin (loading control) are also shown, data represents three independent blots. (B) *Piwil1* and *β-catenin* mRNA levels were also assessed in shRNA-cont and shRNA-*piwil1* cells and the RT-PCR data show down-regulation of both *piwil1* and *β-catenin* mRNA levels which is consistent with the Western blot analysis. RT-PCR experiments were carried out at least 3 independent times.

RASSF1C has been previously linked to the Wnt signaling pathway, as RASSF1C over-expression increases and silencing or RASSF1C decreases β-catenin accumulation in lung cancer cells [16]. We have previously demonstrated that knock-down of RASSF1C expression results in down-regulation of PIWIL1 gene expression [12]. Thus we wanted to determine if PIWIL1 gene knock-down will affect β-catenin expression. Knock-down of PIWIL1 gene expression using a shRNA-lentiviral vector resulted in lower levels of β-catenin mRNA and protein (Figure 6). Our findings suggest that PIWIL1 may modulate β-catenin levels, and that RASSF1C and PIWIL1 may play a key role in Wnt pathway signaling to promote tumorigenesis.

### Intracellular IGFBP-5 appears to modulate RASSF1C function in lung cancer cells

**Figure 7:**
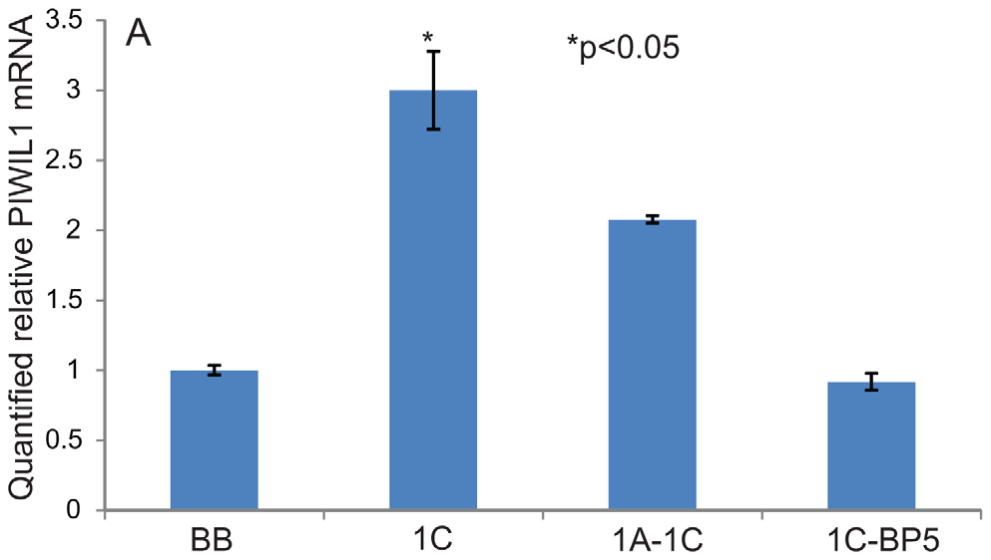
Effect of RASSF1C-IGFBP5 interaction on PIWIL1 mRNA expression. *Piwil1* mRNA expression was assessed in H1299 cells stably transduced with MLV-HA-back bone (BB), MLV-HA-RASSF1C (1C), MLV-HA-RASSF1A and 1C (1A-1C) and MLV-HA-1C and IGFBP-5 (1C-BP5). RT-PCR data show that *piwil1* mRNA expression in cells co-expressing 1C and BP5 was reduced compared to cells over-expressing 1C and 1A and1C. Data is representative of at least 3 independent experiments, and the values represent the mean±SEM. * P<0.05: for 1C compared to BB.

**Figure 8:**
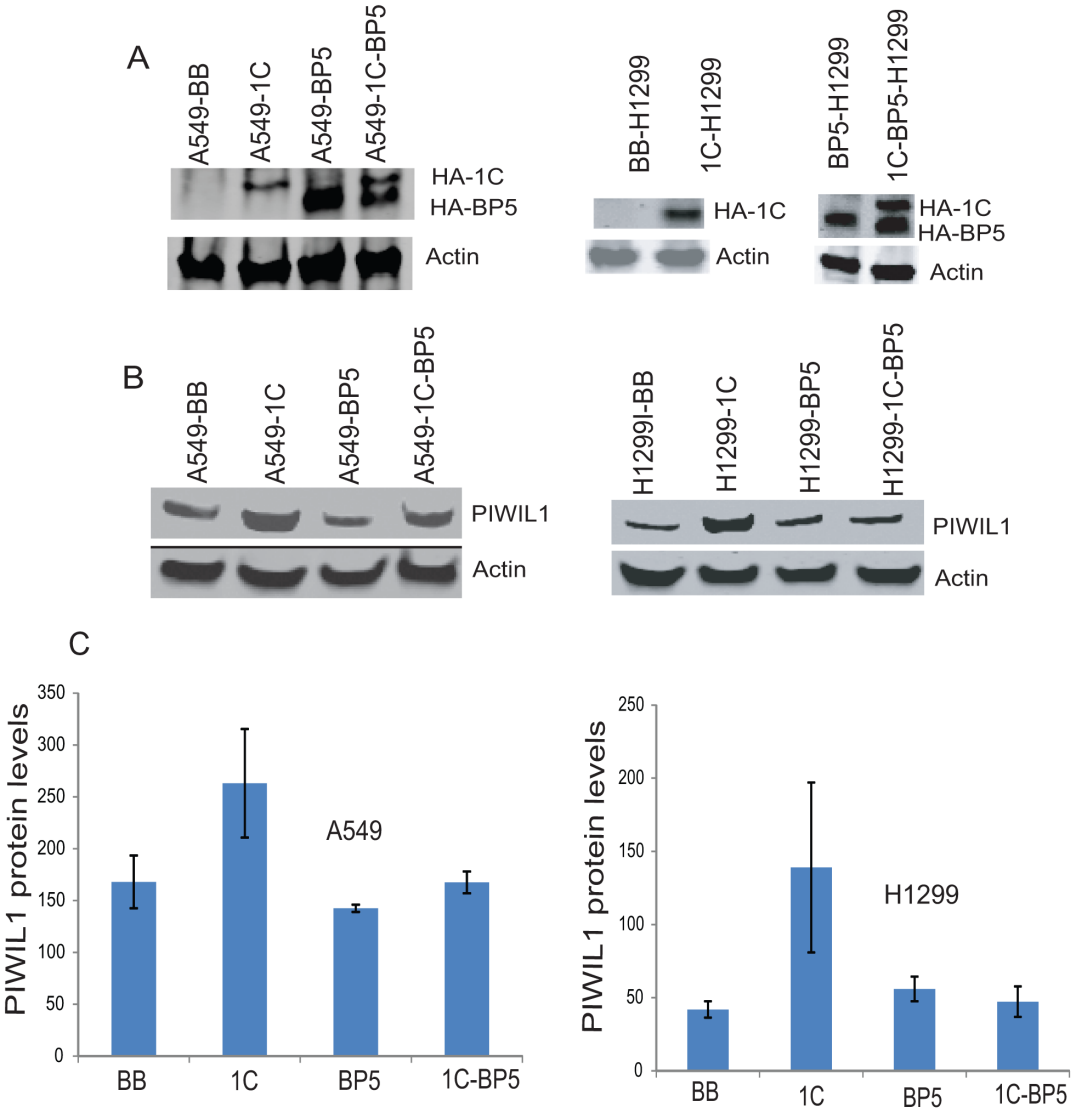
Effect of RASSF1C-IGFBP5 interaction on PIWIL1 protein levels. (A) Western blot analysis of NCI-H1299 and A549 cells stably transduced with MLV-backbone (BB), MLV-HA-RASSF1C (1C), MLV-HA-IGFBP-5 (BP5), and co-transduced with both MLV-HA-RASSF1C and –IGFBP-5 (1C-BP5) and treated with 1 µg/ml doxycycline for 48 h. The anti-HA tag antibody detected a HA-RASSF1C and HA-IGFBP-5 fusion protein in cells that over-expressed each alone and those that co-expressed them both. The fusion protein was visualized using fluorescently labeled secondary antibodies. (B) Western blot analysis of A549 and H1299 lung cancer cells stably transduced with MLV-backbone (A549-BB and H1299-BB), MLV-HA-RASSF1C (A549-1C and H1299-1C), MLV-HA-IGFBP-5 (A549-BP5 and H1299-BP5), and MLV-HA-RASSF1C/MLV-HA-IGFBP-5 A549-1C-BP5 and H1299-1C-BP5). The anti-PIWIL1 antibody was used to probe the Western blot. PIWIL1 is up-regulated in A549 and NCI-H1299 cells over-expressing RASSF1C, but not in cells that express IGFBP-5 or co-express both IGFBP-5 and RASSF1C. (C) Shows normalized PIWIL1 protein levels to actin (loading control) with standard deviations, data represents three independent blots.

**Figure 9:**
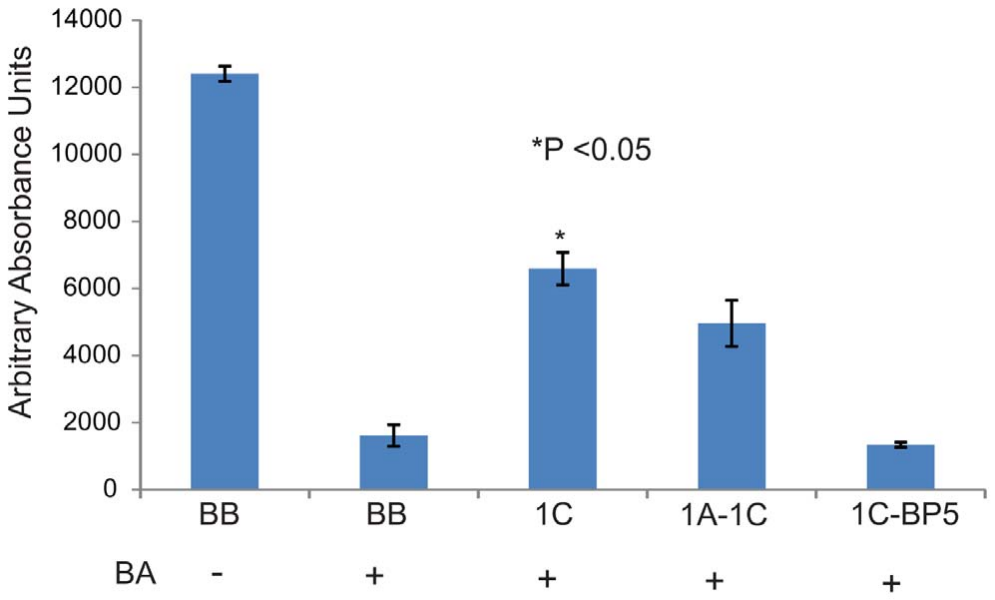
Co-expression of RASSF1C-IGFBP5 sensitizes cells to betulinic acid . H1299-BB, H1299-1C, H1299-1A-1C, and H1299-1C-BP5 cells were treated with betulinic acid (BA) for 24 h. Cells were then assayed for cell viability/proliferation. Cells co-expressing RASSF1C-IGFBP-5 were more sensitive to BA compared to cells over-expressing RASSF1C or co-expressing RASSF1A-RASSF1C. Data is representative of at least 3 independent experiments, and the values represent the mean±SEM. * P<0.05: for 1C compared to BB.

Recent work shows that PIWIL1 over-expression down-regulates a number of tumor suppressor genes, including IGFBP-5, in human mesenchymal stem cells [18]. Previous work in our laboratory has demonstrated that RASSF1C binds to IGFBP-5 and may regulate osteosarcoma cell proliferation and apoptosis [20]. Thus, we wanted to investigate the effect of RASSF1C/ IGFBP-5 interaction on PIWIL1 gene expression in lung cancer cells. Interestingly, we found that co-expression of RASSF1C and IGFBP-5 reduced PIWIL1 mRNA and protein levels in lung cancer cell lines, while co-expression of RASSF1A-RASSF1C did not have a major effect on PIWIL1 mRNA levels compared to cells over-expressing RASSF1C (Figure 7). PIWIL1 protein levels were also reduced in lung cancer cells co-expressing RASSF1C-IGFBP-5 compared to cells over-expressing RASSF1C (Figure 8). Furthermore, we found that lung cancer cells co-expressing RASSF1C and IGFBP-5, but not cells co-expressing RASSF1A and RASSF1C, were as sensitized to BA inhibition as the control cells (Figure 9). We should not that RASSF1A over-expression alone did not enhance the growth inhibitory effects of BA (data not shown). These findings suggest that intracellular IGFBP-5, but not RASSF1A, negatively impacts RASSF1C functions. Linking RASSF1C/IGFBP-5 interaction to the modulation of PIWIL1 gene expression and cell proliferation is a novel finding that requires further investigation.

### Inhibition of ATM-AMPK pathway induces RASSF1C gene expression

**Figure 10:**
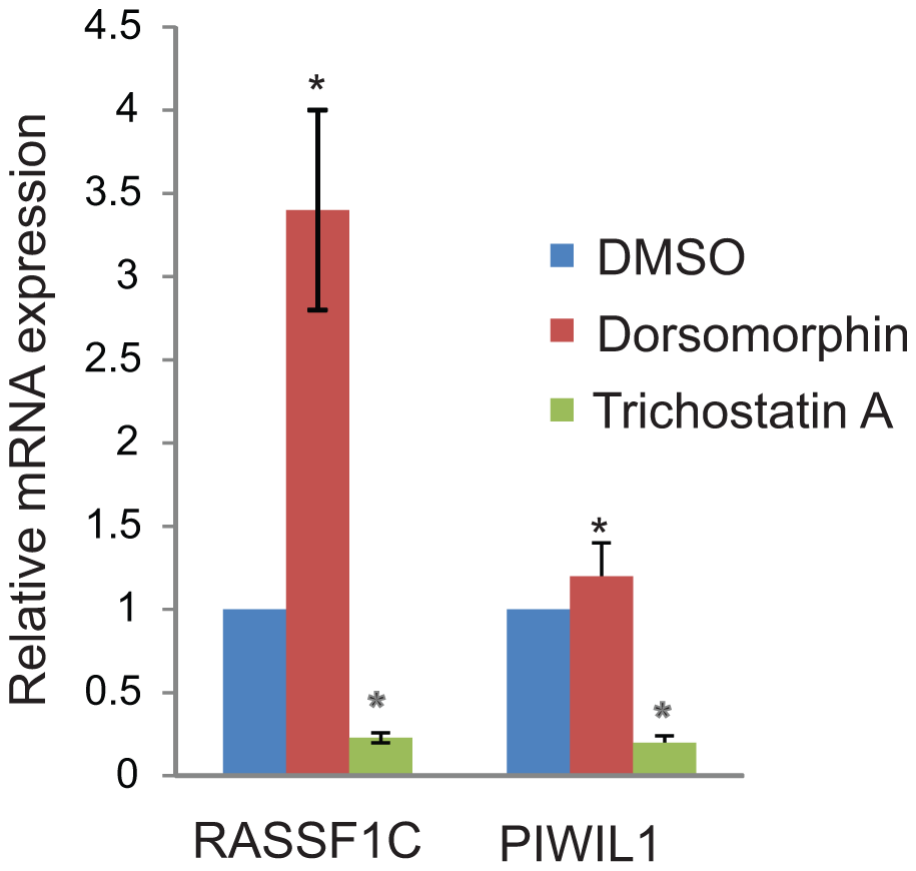
Dorsomorphin and Trichostatin A have opposite effects on RASSF1C and PIWIL1 gene expression. H1299 lung cancer cells were treated with Dorsomorphin and Trichostatin A at a concentration of 10 µM to validate the PCR array data. RT-PCR analysis shows that Dorsomorphin significantly up-regulated RASSF1C gene expression while slightly up-regulating PIWIL1 gene expression. Trichostatin A treatment significantly down-regulated RASSF1C and PIWIL1 mRNA levels. The data presented is an average of three independent RT-PCR experiments done in triplicate, *  =  P<0.05.

Currently nothing is known about the upstream signaling cascades involved in regulating RASSF1C gene expression. Therefore, we performed a transcriptome PCR array study to identify chemical inhibitors and associated signaling pathway(s) that modulate RASSF1C expression. The PCR array consisted of cDNA from the breast cancer cell line MCF7 treated with 90 different chemical inhibitors that regulate various signaling pathways. The array was screened with RASSF1C gene specific primers and several chemical inhibitors that appear to up-regulate and down-regulate RASSF1C expression by ≥ 1.5 fold were identified. The most notable reagents are Dorsopmorphin (AMPK inhibitor) and KU60019 (ATM inhibitor) which up-regulate RASSF1C expression by 2.8 and 2.4 fold respectively; and Trichostatin A (HDAC inhibitor and AMPK activator) down regulates RASSF1C expression by 2 fold (Table 1). We subsequently confirmed the effect of Dorsomorphin and Trichostatin on RASSF1C expression in H1299 lung cancer cells (Figure 10). These findings are novel and suggest that the ATM-AMPK pathway may modulate RASSF1C expression/function in lung cancer cells.

**Table 1:** PCR array screen.

Gene Target	Reagent	RASSF1C mRNA Fold
AMPK	Dorsomorphin	2.8
ATM Kinase	KU60019	2.4
HDAC	Trichostatin A	-2

MCF7 cells were treated with 90 chemical inhibitors and screened with RASSF1C gene specific primers. The PCR array screen identified several chemical inhibitors that up- and down-regulated RASSF1C gene expression. The two top modulators of RASSF1C gene expression are listed. Dorsomorphin (an inhibitor of AMPK) and KU60019 (an inhibitor of ATM kinase) up-regulate and Trichostatin A (an inhibitor of HDAC) down-regulates RASSF1C gene expression. The PCR array data analysis was performed using the Qiagen software program as directed.

## Discussion

RASSF1 appears to be a gene with dual functionality, both as a tumor suppressor protein (RASSF1A isoform), and as an oncoprotein (RASSF1C isoform) [11]–[16]. In this article, we report that over-expression of RASSF1C not only enhances lung cancer cell proliferation, but also promotes cell migration. This is consistent with our previous findings in breast cancer cells [13]. In addition, RASSF1C over-expression also reduces the effects of the anti-cancer agent, betulinic acid, which is known to decrease lung cancer cell proliferation [22]. This further supports the growth promoting and apoptosis attenuating actions of RASSF1C.

We have previously shown that RASSF1C modulates PIWIL1 gene expression, a stem cell self-renewal gene. Hence, it is possible that RASSF1C may contribute to lung cancer stem cell development and progression. Consistent with this, we found that CD133^+^ lung cancer cells (which exhibit cancer stem cell-like characteristics [24], [25] over-expressing RASSF1C appear to form larger and more numerous tumor spheres compared to control CD133^+^ cells. PIWIL1 gene expression is elevated in many human cancer cells, and it has been suggested that PIWIL1 may be involved in tumorigenesis [17], [18], [26]. Recently, sh-RNA *piwil1* gene knock-down in lung cancer stem cells (SSCloAldebr) has been shown to significantly reduce tumor growth in nude mice, further implicating the role of the PIWIL1 protein in maintaining cancer stem cell proliferation [28].

BA has previously been shown to reduce human cancer cell proliferation and down-regulate *piwil1* mRNA expression in gastric adenocarcinoma cells [22], [26]. We assessed the effects of BA on *piwil1* gene expression in lung cancer cells using RT-PCR and Western blot analysis. While treating lung cancer cells with BA resulted in a reduction in cell proliferation (Figure 4) and in down-regulation of the *piwil1* mRNA which is consistent with published literature. The *piwil1* gene is over-expressed in several human cancers, and knock-down of *piwil1* decreases (and over-expression of *piwil1* gene increases) tumor growth *in vivo* [18], [19], [28]. However, very little is known about its role in lung cancer cell growth and progression. We knocked down *piwil1* gene expression to further learn about its functions in lung cancer cells. We found that *piwil1* gene knock-down resulted in a decrease in endogenous β-catenin protein levels. We have shown that RASSF1C up-regulates PIWIL1 expression [12], and others have shown that RASSF1C enhances the accumulation of β-catenin protein levels in A549 lung cancer cells [15]. Our findings point to a potential link between RASSF1C and PIWIL1 and the canonical Wnt signaling pathway, a pathway that plays a key role in not only keeping stem cells in a self-renewing and undifferentiated state, but also in promoting tumorigenesis [19], [28].

PIWIL1 has recently been shown to inhibit differentiation of sarcoma precursors *in vitro* and to induce sarcomas *in vivo* (19). PIWIL1 induces sarcomagenesis through down-regulation of tumor suppressors and cyclin-dependent kinase inhibitors via hypermethylation [19]. One of the tumor suppressors down-regulated by PIWIL1 is IGFBP-5, which we have previously shown to be an interacting partner of RASSF1C [19]. Therefore, we wanted to study the effect of RASSF1C/IGFBP-5 interaction on PIWIL1 gene expression in lung cancer cells. Intriguingly, co-expression of RASSF1C and IGFBP-5 significantly negated RASSF1C up-regulation of PIWIL1 gene expression and also enhanced the inhibitory effects of BA compared to cells that either express RASSF1C or co-express RASSF1A and RASSF1C (Figure 9). Together, these findings suggest that IGFBP-5 may bind and sequester RASSF1C, thus preventing up-regulation of PIWIL1 gene expression by RASSF1C. This linkage of IGFBP-5/RASSF1C interaction with modulation of PIWIL1 expression is a novel finding and deserves further study and validation.

Currently nothing is known about how RASSF1C gene expression is regulated, and thus we performed a transcriptome PCR array screen and identified several chemical inhibitors that appear to modulate RASSF1C gene expression. Among these chemical inhibitors, we found that the chemical inhibitor Dorsomorphin (also known as compound C) and KU60019 up-regulate RASSF1C > 2 fold while the chemical Trichostatin A down-regulates RASSF1C gene expression by 2 fold in breast and lung cancer cells (Table 1). Dorsomorphin has been shown to inhibit the effects of ionizing radiation (IR) on cell cycle arrest and cell proliferation through the inactivation of ATM-AMPK-p21^waf/cip^ pathway [29]. Activation of ATM-AMPK-p21^waf/cip^ pathway with Metformin has been recently shown to inhibit growth and to enhance IR effects on NSCLC cells [30]. Trichostatin A has also been shown to activate AMPK, to inhibit HDAC, to arrest cell growth, and induce apoptosis in human cancers [31]. ATM has been shown to phosphorylate the RASSF1A isoform in response to DNA damage leading to MST2 and LATS phosphorylation/activation and p73 stabilization [32]. We recently have shown that RASSF1A and RASSF1C have opposite effects on cell proliferation and apoptosis, and that RASF1C had no effect on MST1/2 phosphorylation [15]. Hence it will be interesting to determine if inhibition of ATM kinase and the associated induction of RASSF1C gene expression will enhance lung cancer cells’ resistance to DNA damage response leading to genetic instability and oncogenesis.

## Conclusions

**Figure 11:**
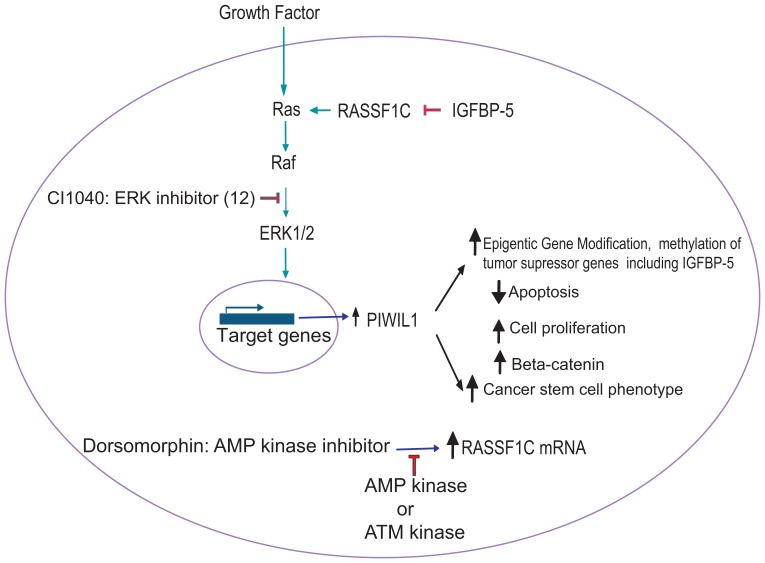
Proposed RASSF1C mechanistic pathway for lung cancer growth and progression. This model is based on our (12) and others previous published work and current data presented in this article. RASSF1C activates the MEK-ERK1/2 pathway (12), which controls a wide variety of genes that promote cell division and proliferation. Over-expression of RASSF1C up-regulates Piwil1 gene expression (12). Treatment of H1299 lung cancer cells with the MEK-ERK1/2 pathway inhibitor CI1040 resulted in down-regulation of Piwil1 mRNA levels (12). PIWIL1 is widely over-expressed in tumors compared to normal tissue, and it may have important functions in cancer initiation, maintenance, or progression (increased epigenetic gene modification, proliferation and cell migration, and reduced apoptosis). PIWI-like proteins have been shown to interact with PIWI-interacting RNAs (piRNAs) to form complexes that regulate transcriptional and translational repression leading to inhibition of apoptosis, stimulation of cell division and proliferation, and down-regulation of cyclin inhibitors and tumor suppressors (18). Over-expression of PIWIL1 down-regulates IGFBP-5 (18), which is an interacting partner and inhibitor of RASSF1C (19). Thus, co-expression of RASSF1C and IGFBP-5 abrogates the up-regulation of PIWIL1 expression in response to increased RASSF1C, suggesting that IGFBP-5 could block RASSF1C actions through the ERK1/2 pathway resulting in reduced PIWIL1 expression and function. Also, RASSF1C mRNA expression is regulated by AMP and ATM kinases. Various key components of the proposed RASSF1C pathway will be tested to validate this model.

We present novel findings potentially linking RASSF1C and PIWIL1 to the Wnt signaling pathway and lung tumorigenesis. We also show that IGFBP-5 may be an important molecule for modulating RASSF1C/PIWIL1 effects in lung cancer. The IGFBP-5, RASSF1C, and PIWIL1 genes may constitute a new axis in controlling lung cancer cell development and progression. Our findings provide a potential mechanism linking RASSF1C to the ATM-AMPK-p21^waf/cip^ pathway. Various key components of a proposed RASSF1C pathway are being tested to validate our working model as shown in Figure 11.
